# Space-flown spearmint (*Mentha spicata* L.) seeds potentially tolerate nitro-oxidative stress from plasma-activated water

**DOI:** 10.3389/fpls.2026.1836606

**Published:** 2026-07-29

**Authors:** Claude Simon A. Naco, Noel S. Quiming, Christine Joy M. Octavo, Ciara Christianne Y. Lim, Jasper John A. Obico, Aldrin P. Bonto, Rodolfo E. Sumayao, Maria Carmen S. Tan, Michael Dominic M. Ajero, Bryndell J. Alcantara, Aldrin A. Tan, Kathrina Lois M. Taaca, Magdaleno R. Vasquez, Julius Andrew P. Nuñez

**Affiliations:** 1 Laboratory of Materials, Department of Physical Sciences and Mathematics, College of Arts and Sciences, University of the Philippines Manila, Manila, Philippines; 2 Natural Products and Peptidomics Laboratory, Department of Physical Sciences and Mathematics, College of Arts and Sciences, University of the Philippines Manila, Manila, Philippines; 3 Department of Physical Sciences and Mathematics, College of Arts and Sciences, University of the Philippines Manila, Manila, Philippines; 4 Department of Biology, College of Arts and Sciences, University of the Philippines Manila, Manila, Philippines; 5 Department of Chemistry, De La Salle University Manila, Manila, Philippines; 6 Plasma-Material Interactions Laboratory, Department of Mining, Metallurgical and Materials Engineering, College of Engineering, University of the Philippines Diliman, Quezon City, Philippines

**Keywords:** 2,4-Di-tert-butylphenol, abiotic stress resistance, Ar plasma, space agriculture, spearmint germination

## Abstract

Establishing fresh food sources during exploratory missions requires the identification of compatible plant species and accompanying technologies needed to support their growth in the space environment. Here, we investigated the use of plasma-activated water (PAW), generated via cold atmospheric pressure plasma, as a non-chemical fertilizer to aid the cultivation of *Mentha spicata* L. (spearmint) seeds previously flown aboard the International Space Station for 6 months. This study showed that PAW generated at a 10-min discharge (PAW10) may become inhibitory to plant development due to its complex chemistry, including the excessive amounts of nitrates (NO_3_^-^) introduced during the long-duration treatment. This was reflected in the reduced germination and growth characteristics observed in PAW10-treated seeds following a 14-day germination assay. These results were not observed when PAW was activated for only 5 min (PAW5), potentially indicating a dose-dependent response to the plasma-induced reactive oxygen and nitrogen species (RONS) in the water treatment. However, GC-EI/MS revealed an alteration in the non-enzymatic antioxidant response in space-flown seeds treated with PAW10, where a decrease in 2,4-di-tert-butylphenol (2,4-DTBP), a phenolic defense compound, was seen. We speculate that, if not through non-enzymatic mechanisms, the apparent resistance against PAW10 toxicity may have risen from a shift towards enzymatic defenses against nitro-oxidative stress, a common phenomenon reported in other plants. Further study is required to confirm this hypothesis. Overall, the present work raises spearmint as a promising candidate for space cultivation, or at the very least, a model to study the plant stress response in space. Notably, prior space exposure conferred a greater tolerance against nitro-oxidative stress in spearmint, mitigating the adverse effects associated with excessively activated PAW.

## 1 Introduction

To support astronauts’ living conditions, spacecraft are equipped with life support systems (LSSs) that provide the following functions: maintaining pressure, temperature, and humidity; giving useable water and breathing air; delivering food; and handling waste (Jha et al., 2023). Currently available LSSs are mainly open systems that use a variety of physicochemical processes with limited recycling capabilities that are unable to independently eliminate waste and produce a sustainable food and oxygen supply (Kabashkin and Glukhikh, 2023). Through the coordination of other biological components such as plants and microorganisms, spacecraft can be equipped with self-sustaining functions for O_2_-CO_2_ balance (via photosynthesis and respiration), water recycling (plant transpiration can be recovered by condensation and directed into water recovery systems), food production (plant products can be prepared into meals), and organic waste management (via biodegradation) (Arroyo et al., 2023). The integration of these bioregenerative LSSs (BLSSs) is still being tested in space conditions through the following initiatives: Micro-Ecological Life Support System (MELiSSA) (Lasseur et al., 2010), BioMed-1 (Nelson et al., 2010), Veggie (Ehrlich et al., 2017), MOSPECS (Nelson et al., 2010), etc.

Space agriculture has long been challenged by reduced germination rates and shorter plants. This has been extensively shown across several seed storage experiments in Low-Earth Orbit (LEO) of *Eruca sativa* (Chandler et al., 2020), *Oryza sativa* (Zeng et al., 2021), *Zea mays* (Mei et al., 1994)*, Arabidopsis thaliana* (Zimmermann et al., 1994; Tepfer et al., 2012), and *Medicago sativa* (Ren et al., 2010). Therefore, an efficient BLSS requires the very careful selection of plants based on the following criteria: they must (a) occupy minimum space, (b) provide high nutritive value, (c) be easily cultivated, and (d) exhibit resistance to space factors (De Micco et al., 2023b, 2023a). In this regard, herbs are excellent candidates, as they meet the aforementioned criteria for extraterrestrial cultivation. Of particular interest is the genus *Mentha* (mint), which has been increasingly gaining recognition for its potential uses in BLSSs (Arroyo et al., 2023; Cheng et al., 2025; Marshall Porterfield et al., 2025). It spans about 30 species of perennial aromatic herbs with several inter-specific hybrids and cultivars, all of which are characterized by their physiological adaptability, biochemical efficiency, and economic value (El Boukhari and Fatimi, 2025; Mahdavian, 2025; Naseem et al., 2025). At the very least, they are a practical model for studying the capacity of plants to persist against the harsh conditions of space, largely due to the fact that they can be grown from seeds with a short experimental timeline, with measurable physiological and metabolic responses across a variety of environments (Zaki et al., 2024; Shelepova et al., 2025). Moreover, in spite of their small stature of 10 to 20 cm, they remain rich in phenolic constituents that not only enhance the nutritional values of foods but also possess high antioxidant activity that may confer a greater tolerance to the oxidative stress induced by space-radiative processes (Tafrihi et al., 2021; Saqib et al., 2022; Yousefian et al., 2023).

Aside from plant selection, another consideration for building BLSSs is assistive technologies that remedy the apparent trend of hindered germination and growth in space-flown seeds; non-thermal plasma (NTP) treatments show promise for this purpose (Priatama et al., 2022). When air is ionized by electric fields, the resulting plasma gives rise to high-energy radiation that, in turn, drives the formation of reactive oxygen and nitrogen species (RONS). Acute exposure to RONS and their derivatives can trigger biological processes in seeds, such as dormancy alleviation and root/shoot development (Waskow et al., 2021). This approach is preferred over other methods due to its low maintenance, cost-efficiency, and involvement of species that are non-toxic, found in nature, and short-lived. Additionally, NTP does not penetrate seeds deep enough to injure any cells, making it a promising strategy for enhancing seed germination after exposure to elevated levels of space radiation. NTP treatment of seeds can be achieved through direct contact with plasma or indirectly through exposure to plasma-activated species dissolved in water. This plasma-activated water (PAW), when generated under atmospheric conditions, is known to contain a high nitrate and nitrite content. PAW can then be considered as a fertilizer, given nitrogen’s role as the backbone of metabolic processes that directly affect plant growth (Graves et al., 2019). In fact, previous studies on PAW report the effectiveness of this treatment in improving the seed germination and plant growth of mung beans (Chou and Ting, 2023), thale cress (Ka et al., 2021), and tobacco (Lee et al., 2020).

The current study investigated the effects of PAW treatment on space-flown spearmint (*Mentha spicata*) seeds to provide insight into the potential use of this herb and respective treatment in BLSSs, especially since seed cultivation of *Mentha* species already yields low germination percentages without any enhancers (Elhindi et al., 2016). This is the first study to investigate the use of PAW technology for potential integration in BLSSs. These findings also contribute to the limited literature on seed storage experiments in space by highlighting spearmint as a candidate herb that can withstand the harmful conditions of the space environment.

## 2 Materials and methods

### 2.1 Spearmint seeds

#### 2.1.1 Procurement and maintenance

The space-flown (S) spearmint seeds used in this study were obtained from the Japan Aerospace Exploration Agency (JAXA) and through the Philippine Space Agency (PhilSA), a collaborator on JAXA’s Asian Herb in Space program. The seeds were launched into the Kibo module of the International Space Station via the SpaceX Dragon spacecraft during the CRS-21 mission (SpX-21) on December 7, 2020. During spaceflight, the seeds were enclosed in sealed plastic pouches and shielded from sunlight; however, they were not protected from high-energy particles. Otherwise, other environmental conditions within the Kibo module were maintained to approximate those at Earth’s sea level. The seeds were returned to Earth on June 4, 2021, via the CRS-22 mission (SpX-22), which landed near the coast of Florida, USA. Shortly thereafter, the seeds were transferred to Japan. Concurrently, non-space-flown (NS) seeds had been stored at the Tsukuba Space Center in Japan under conditions like those of the Kibo module, only differing in terms of exposure to microgravity and high-energy radiation.

Prior to their transfer to the Philippines, both the S and NS seeds were treated with 0.4% captan (fungicide) and 0.3% acephate (insecticide) as part of the standard procedure by Japan’s Ministry of Agriculture, Forestry, and Fisheries (MAFF) Plant Protection Station. On March 8, 2024, both seed cohorts were acquired from the PhilSA for the present study. Sample size and compartmentalization were determined by the management of the project; specifically, the weight of seeds received amounted to approximately 1.0 g per group, roughly corresponding to 2000 seeds each. Throughout experimentation, they were kept in their original plastic containers and stored with silica gel beads at 0-4 °C. During transport, as needed, seeds were kept over ice packs inside insulated coolers.

#### 2.1.2 Viability test via tetrazolium staining

Upon receipt, both S and NS seeds were tested for viability according to the protocol for seeds under the *Lamiaceae* family (Robinson et al., 2024). One hundred seeds from each cohort were analyzed as follows. Seeds were first soaked in deionized water for 24 h. Afterwards, they were removed from the soak and fixed in place using double-sided tape and sliced through the round, wider end using a stainless-steel razor blade to expose the cotyledon. The larger portions of the sliced seeds were transferred to microcentrifuge tubes, where they were fully submerged in a 1% solution of 2,3,5-triphenyl tetrazolium chloride (pH 6.5 to 7.5). After soaking for 48 h, the seeds were removed from the solution and visualized under a stereomicroscope to observe staining. A fully pink or bright red stain indicates a viable seed; partially stained seeds are considered viable but may produce abnormal seedlings; pink or grayish red stains indicate dead tissue; and completely unstained seeds are non-viable.

### 2.2 Atmospheric pressure plasma jet system

#### 2.2.1 Setup

**Figure 1:**
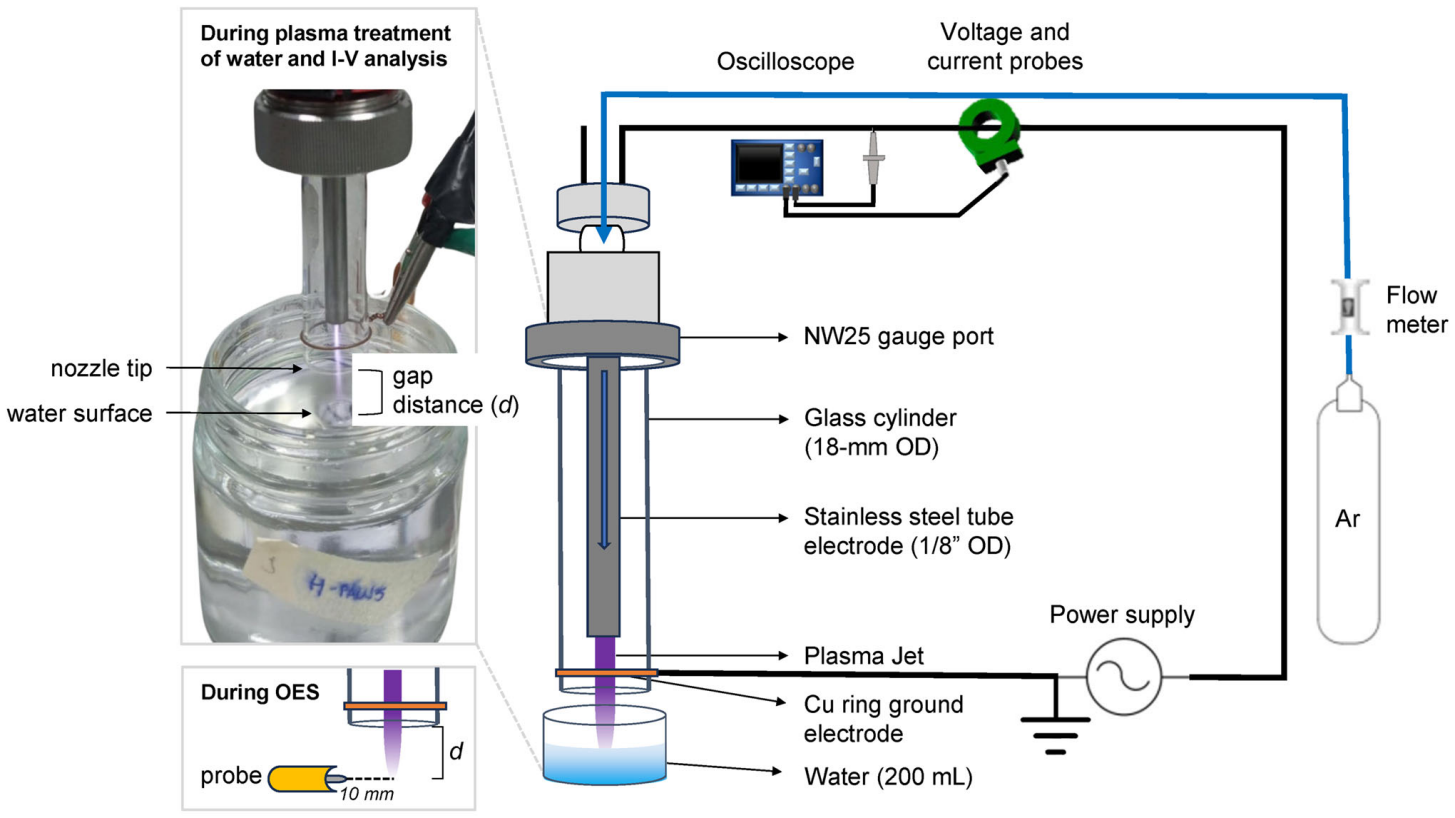
Custom-built atmospheric pressure plasma jet (APPJ) system.

The schematic of the atmospheric pressure plasma jet (APPJ) system used in this study can be found in **Figure 1**. The APPJ nozzle consists of an 18-mm OD borosilicate glass (Pyrex) body secured by an insulated NW25 gauge port, which is held in place by an iron clamp and stand. Inside the nozzle, a 1/8-inch OD stainless steel tube, secured by an Ultra-Torr (Swagelok), is connected to a 99.99% argon (Ar) gas tank. The nozzle contains a copper wire at its mouth to serve as the ground electrode.

Ar plasma was ignited using an alternating current (AC) power source connected to a 15 kV transformer. The input to the power source was controlled by a variac. The gas flow was set to 10 liters per minute (lpm) using a flowmeter, and the variac voltage was set to 40 V.

#### 2.2.2 Waveform analysis

The voltage and current characteristics of the APPJ setup were taken during plasma treatment of water. In doing so, a gap distance between the nozzle tip and water surface was fixed at 1.5 cm (**Figure 1**), as rationalized in the Results section. Both voltage and current waveforms were recorded simultaneously using the PINTEK HVP-40 high-voltage probe with a 1000:1 attenuation ratio and a PEARSON™ current probe with a sensitivity of 1 V/A, respectively. Signals from these probes were fed to the DS1M12 Stingray USB oscilloscope (EasySync, Ltd) and analyzed using the EasyScope II application.

#### 2.2.3 Plasma characterization via optical emission spectroscopy (OES)

The Ocean Optics HR4000CG-UV-NIR spectrometer and its accompanying fiber optic cable were used to analyze the excited reactive species in the generated plasma plume when it reaches the water interface. In doing so, the water was removed from the setup to allow the fiber optic cable to be positioned 10 mm away from the plume in a perpendicular manner (**Figure 1**). Spectra were then acquired at *d* = 1.5 cm using a 1500 ms integration time between 190 and 1150 nm. Peaks were validated by comparison with the NIST Atomic Spectra Database (Kramida et al., 2024).

### 2.3 Plasma-activated water (PAW)

#### 2.3.1 PAW generation

Two hundred milliliters of deionized water (ATR Trading System, Quezon City, PH) were placed in a 250-mL borosilicate glass jar and autoclaved at 121 °C for 15 min at 15 psi. After cooling, plasma-activation of water was performed for 5 min (PAW5) and 10 min (PAW10) to generate PAW treatments, while untreated sterile deionized water (DW) was kept as a control. In doing so, the APPJ nozzle was positioned directly above the water surface at d = 1.5 cm. The treatment durations were selected based on similar PAW agar studies where activation times around this range were used (Lee et al., 2020; Ka et al., 2021). Throughout both treatment times, no reduction in water volume via spillage of water nor evaporation was observed.

All subsequent handling of water samples was performed under an alcohol lamp, unless otherwise stated, to minimize microbial contamination.

#### 2.3.2 Physicochemical measurements

Immediately prior to the germination assay, the pH of the water samples was measured using the Hanna edge^®^ Digital pH Meter - HI2002–01 at room temperature. Also, a qualitative test for the presence of nitrates and nitrites was performed using the YIERYI^®^ 14-in-1 Reagent Strips for Water.

Freshly generated PAW treatments and DW were also submitted in triplicate to ELARSI (Quezon City, PH) for nitrate quantitation using an ion-selective electrode (Cole-Parmer^®^). These samples were not used for the germination assay and were analyzed on the same day as plasma activation.

### 2.4 Germination assay

#### 2.4.1 Agar preparation

To provide a germination substrate, the seeds were placed on a Petri dish with agar made of the treated water. The agar solution was made following the protocol of Lee et al. (2020) with slight modifications. Within two hours after plasma activation, all water samples were adjusted to pH 6.9 to 7.0 with 1 M KOH and 1 M HCl solution. Afterwards, agar powder (TM media, Agar Agar, Type I) was added to make 1.5% solutions, which were melted in the microwave (Midea FP 61MMVO20LMTL W1). When slightly cooled, the agar preparations were supplemented to 90 mm × 15 mm sterile plastic Petri dishes under a BSC II.

#### 2.4.2 Seed preparation and germination

Uniformly sized and colored seeds that showed no signs of damage (e.g., shed coat, crushed appearance, etc.) were selected. These seeds were surface-sterilized according to the protocol of Elhindi et al. (2016), with slight modifications; they were soaked sequentially in 5% (v/v) sodium hypochlorite for 10 min, followed by 70% (v/v) ethanol for 1 min, and then in sterile deionized water for 1 min. Afterwards, the seeds were sown on agar and incubated at 26 °C until analysis (MRC Labs TOU-50-Series BenchTop Orbital Shaker Incubator).

### 2.5 Germination and growth characteristics

To assess the germination and growth of spearmint over PAW agar, seedlings from a 14-day incubation period (ten seeds per plate for each experimental group) were analyzed for five trials. The number of germinated seedlings was monitored every other day up to 14 days, wherein a seed was considered to have germinated when the emerging radicle became visually longer than the longest axis of the seed. At the end of the germination assay, the characteristics of each group were measured (**Table 1**), as adapted from the germination study by Langa et al. (2024). Seedling length was measured using a vernier caliper; however, estimations using IMAGEJ were performed for seedlings below 1 mm (Schneider et al., 2012).

**Table 1:** Characteristics measured after 14-day germination.

Index	Equation	Units	Purpose
Seedling length (SL)	$SL=\frac{\sum length\;at\;Day\;14}{N_{14}}$	cm	required for vigor index
Final germination percentage (FGP)	$FGP=\frac{N_{14}}{N}\times 100\%$	%	to measure capacity to break dormancy
Germination rate index (GRI)	$GRI=\sum_{i=2,4,6,\ldots }^{14}\frac{N_{i}}{i}\;$	counts per day	to measure the speed of germination overtime
Vigor index (VI)	$VI=\frac{SL\times FGP}{100}$	none	to evaluate germination and growth simultaneously

N is the total number of seeds sowed in the experimental group; N_i_ is the number of germinated seeds in the experimental group to have germinated on the i^th^ day.

### 2.6 Gas chromatography-electron ionization mass spectrometry (GC-EI/MS)

Per experimental group, 40 seeds were sown across two plates and left to germinate for 5 weeks to achieve sufficient biomass for extraction and GC-EI/MS. After the incubation period, both ungerminated seeds and germinated seedlings (including roots, shoots, and leaves) were freeze-dried separately, then suspended in 500 μL 80% (v/v) ethanol. The extraction process was then facilitated in an ultrasonic bath at 25 °C for 30 min, followed by brief agitation with a vortex-mixer. Supernatants were collected following centrifugation (13,000 rpm for 20 min), dried using the Standard CentriVap Vacuum Concentrator (Labconco, Kansas City, MO), and resuspended in 250 μL 1:1 DCM: MeOH. Extracts were stored at -18 °C until GC-EI/MS analysis.

GC-EI/MS was employed on the Agilent gas chromatograph-mass spectrometer 7890B system. Vaporizable components were determined using an HP-5ms (5% phenyl methylsiloxane) Ultra Inert column (30 m × 250 mm × 0.25 mm) and ultra-pure helium gas as the mobile phase, where the parameters of helium gas were automated to the stipulated parameters: flow rate of helium gas was at 1 mL/min, pressure fixed at 8.2 psi, with a mean velocity of 36.62 cm/s, and holdup time of 1.37 min. The splitless inlet was maintained at 250°C at 8.2 psi, with an overall flow speed of 24 mL/min, and a septum purge flow speed of 3 mL/min. The injector temperature was set at 250°C. The temperature gradient program was initially at 70°C, and the preset stepwise increments of 2°C/min were achieved until the maximum of 135°C was accomplished, and this temperature was kept for a further 10 min. After the hold period, an additional temperature increment of 4°C/min was implemented to attain the required temperature of 220°C, after which, this temperature was retained for 10 min. The temperature was then augmented to 270°C at a rate of 3.5°C/min, and this fixed temperature was maintained for 37 min. Compound identification was performed via the NIST library 2.0 and percent peak area average was determined from the subsequent total ion chromatograms. Relative abundances of identified compounds were taken by normalizing the individual peak areas to the total chromatogram area.

### 2.7 Sample disposal

After analysis, all seeds and seedlings from the germination assays and extraction were incinerated and disposed of.

### 2.8 Statistical analysis

Preliminary tests of variance homogeneity (Levene’s) and normality (Shapiro-Wilk) were conducted on all data sets. This was followed by either Student’s t-test (seed viability), or one-way (PAW physicochemical properties) or two-way analysis of Variance (germination characteristics). Multiple comparisons were employed by using the *post-hoc* Tukey’s method.

## 3 Results

### 3.1 Ar plasma characteristics

**Figure 2:**
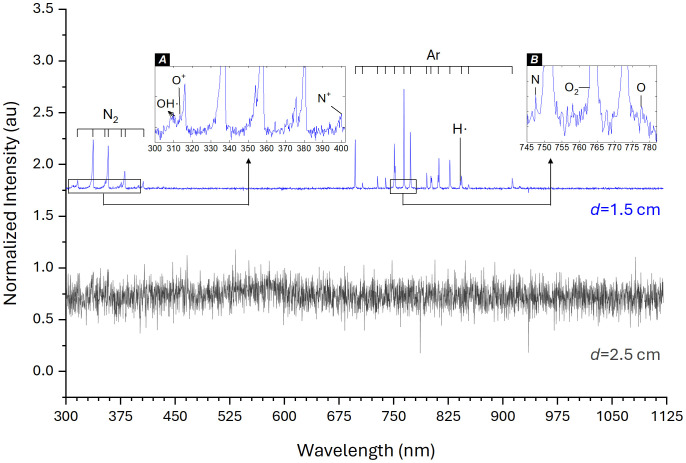
Normalized optical emission spectra of Ar plasma at varying gap distances between the APPJ nozzle tip and water surface (*d* = 1.5 cm and *d* = 2.5 cm). Insets **(A, B)** zoom into different regions of the 1.5-cm spectra to highlight less intense and poorly resolved peaks.

While directing the plume towards the water, it was determined that a gap distance (*d*) of at least 1.5 cm between the nozzle tip and the water surface was required to ensure that no high-current-density arcs arose. Otherwise, abnormal localized discharges would hinder stable plasma operation (Wang et al., 2023a). To determine whether longer gap distances still produced the desired species, optical emission spectra of the Ar plasma were taken at different positions along the length of the plume, beginning from *d* = 1.5 cm (**Figure 2**).

At *d* = 1.5 cm, transition lines of the following major species were observed: N_2_ (337 nm, 357 nm, 375 nm, 380 nm, 406 nm) and Ar (various lines from 700 to 900 nm) (Acharya et al., 2023; Gaur et al., 2023). Molecular N_2_ and Ar are expected to predominate in the spectrum because nitrogen is the main component of the ambient atmosphere and since Ar was used as the feeder gas. Less intense signals were also detected, many of which are indicated in Inset A: OH· (308 to 311 nm), O^+^ (313 nm, 375 nm), N^+^ (400 nm), and H· (841 nm) (Wenåker, 1990; Neuhäuser et al., 1999; Almuslet and Salih, 2012; Li et al., 2013).

Remaining emissions are found within the Ar-dense region but are obscured by neighboring lines (Inset B). This is most apparent with the O_2_ transition at 762 nm, which appears poorly resolved from the main emission peak of Ar at 763 nm. This is exacerbated by the fact that the neutral oxygen molecule does not readily show an emission spectrum. Nonetheless, previous studies have reported this line in atmospheric discharges (Pearse and Gaydon, 1941). In contrast, atomic O was detected through the characteristic line at 777 nm with better resolution from neighboring peaks but with a very weak intensity (Northway and Fry, 1980; Jia et al., 2008; Adhikari et al., 2021). Similarly, we detected a weak line of atomic N at 746 nm (Northway et al., 1980; Musielok et al., 1995). Its relatively weak signal can be explained by N-atom recombination into N_2_ at early regions of the plume, as discussed by a previous study by Chen and Li (2017). They also demonstrated that atomic N can be transported over long distances in the plume by gas flow. Therefore, in this present study, it is possible that N concentrated at a region beyond the measuring distance of *d* = 1.5 cm, and the fiber optic cable could not capture the 746-nm emission of the entire population.

These emission features reflect the production of excited plasma species and can be attributed to Equations 1–12. It is worth noting that while the Ar feeder gas is inert, it participates in the production of reactive species. This is indicated by the 811-nm Ar emission (see **Figure 2**), which is related to Ar metastable states (Qu et al., 2019). These metastable states increase electron temperature and plasma density, thereby paving the way for downstream reactions to proceed.

(1) $$Ar+e^{-}\to Ar^{m}+e^{-}$$

(2) $$Ar^{m}+e^{-}\to Ar^{*}+e^{-}$$

(3) $$H_{2}O+Ar^{*}\to OH•+H•+Ar$$

(4) $$H_{2}O+e^{-}\to OH•+H•+e^{-}$$

(5) $$H_{2}O+e^{-}\to H_{2}O^{+}+2e^{-}$$

(6) $$H_{2}O^{+}\to OH•+H^{*}$$

(7) $$O_{2}+Ar^{m}\to O^{*}+O^{*}+Ar$$

(8) $$O_{2}+e^{-}\to O^{*}+O^{*}+e^{-}$$

(9) $$O+e^{-}\to O^{+}+2e^{-}$$

(10) $$N_{2}+e^{-}\to N^{*}+N^{*}+e^{-}$$

(11) $$N+e^{-}\to N^{+}+2e^{-}$$

Initially, an electron impacts Ar to produce the metastable Ar^m^ (Equation 1). The latter is capable of being excited by an electron (Equation 2), and the excited Ar* can react with water to form reactive OH radicals (Equation 3) (Asghar and Galaly, 2021; Saktioto et al., 2023). OH radicals can also be produced either through the single-step electron-impact dissociation of H_2_O (Equation 4) or the two-step process involving electron-impact ionization of H_2_O followed by dissociation of H_2_O^+^ (Equations 5, 6) (Lee et al., 2020). The H_2_O involved in these two reactions originates from adsorbed water molecules on the electrode of the nozzle, which accumulates when the torch is not in operation (Song et al., 2021). On the other hand, other oxygen species are derived from electronic events involving molecular oxygen (O_2_). Atomic oxygen (O) is produced from the Ar^m^- or electron-impact dissociation of O_2_ (Equations 7, 8), while subsequent ionization of O generates O^+^ (Equation 9) (Mieno et al., 1996; Qu et al., 2019). Similarly, nitrogen species in the plasma are derived from molecular nitrogen (N_2_) from the ambient air. In this light, N^+^ is produced in a similar process as O^+^ production, where N_2_ undergoes dissociation and subsequent ionization (Equations 10, 11) (Dutuit et al., 2013).

The signals produced from all these reactions disappeared at *d* = 2.5 cm. This alludes to the possibility that insufficient molecular collisions occur towards the end of the length of the plume, which could translate to poorly activated water (Taaca et al., 2023). Therefore, a gap distance of 1.5 cm was maintained during PAW generation to appropriately introduce desired nitrogen species into the water, the reaction mechanism of which will be discussed in a later section.

### 3.2 Waveform analysis

**Figure 3:**
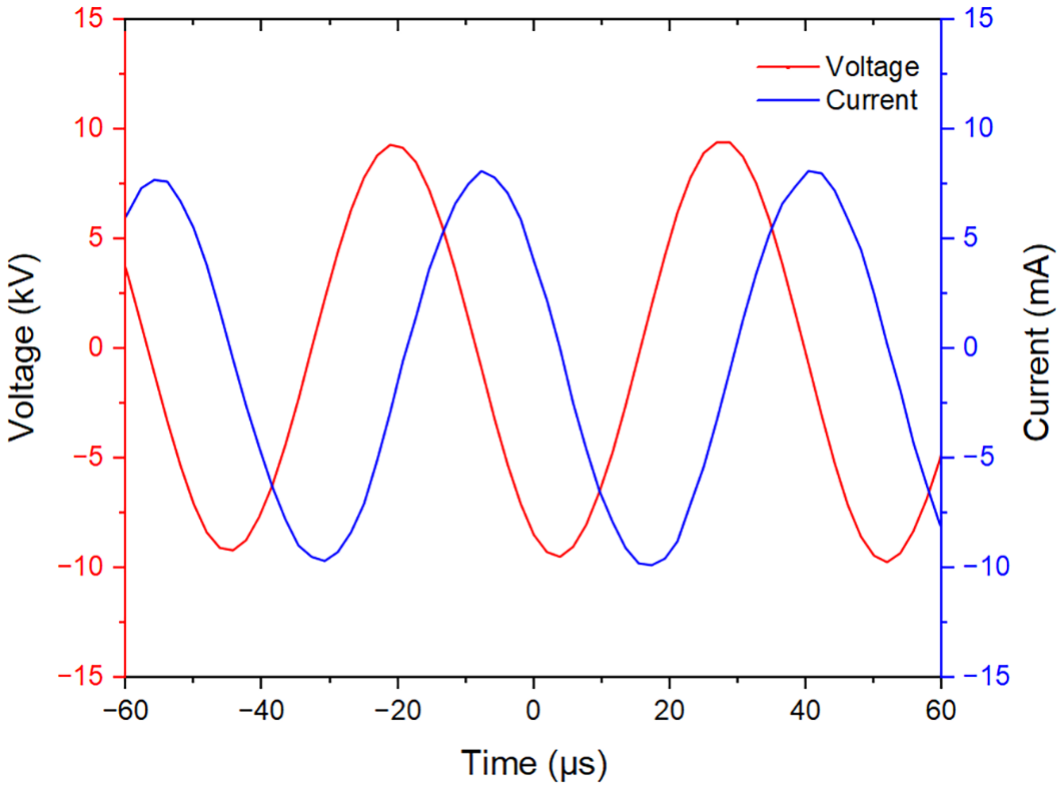
Voltage and current waveforms of Ar plasma.

Waveforms of voltage and current of the Ar plasma discharge are shown in **Figure 3**. At 10 lpm and *d* = 1.5 cm, the waveforms indicate that the discharge exhibited stable behavior, with a peak-to-peak voltage of around 19 kV_p-p_ and a peak-to-peak current of approximately 19 mA_p-p_. The frequency was measured to be around 20 kHz, and the maximum power deposited to the plasma system during water treatment was 73.92 W (determined by multiplying voltage and current values at each timepoint). A 17-μs time lag between the discharge voltage and current indicates that the discharge impedance was capacitive.

### 3.3 Plasma-activated water characteristics

**Figure 4:**
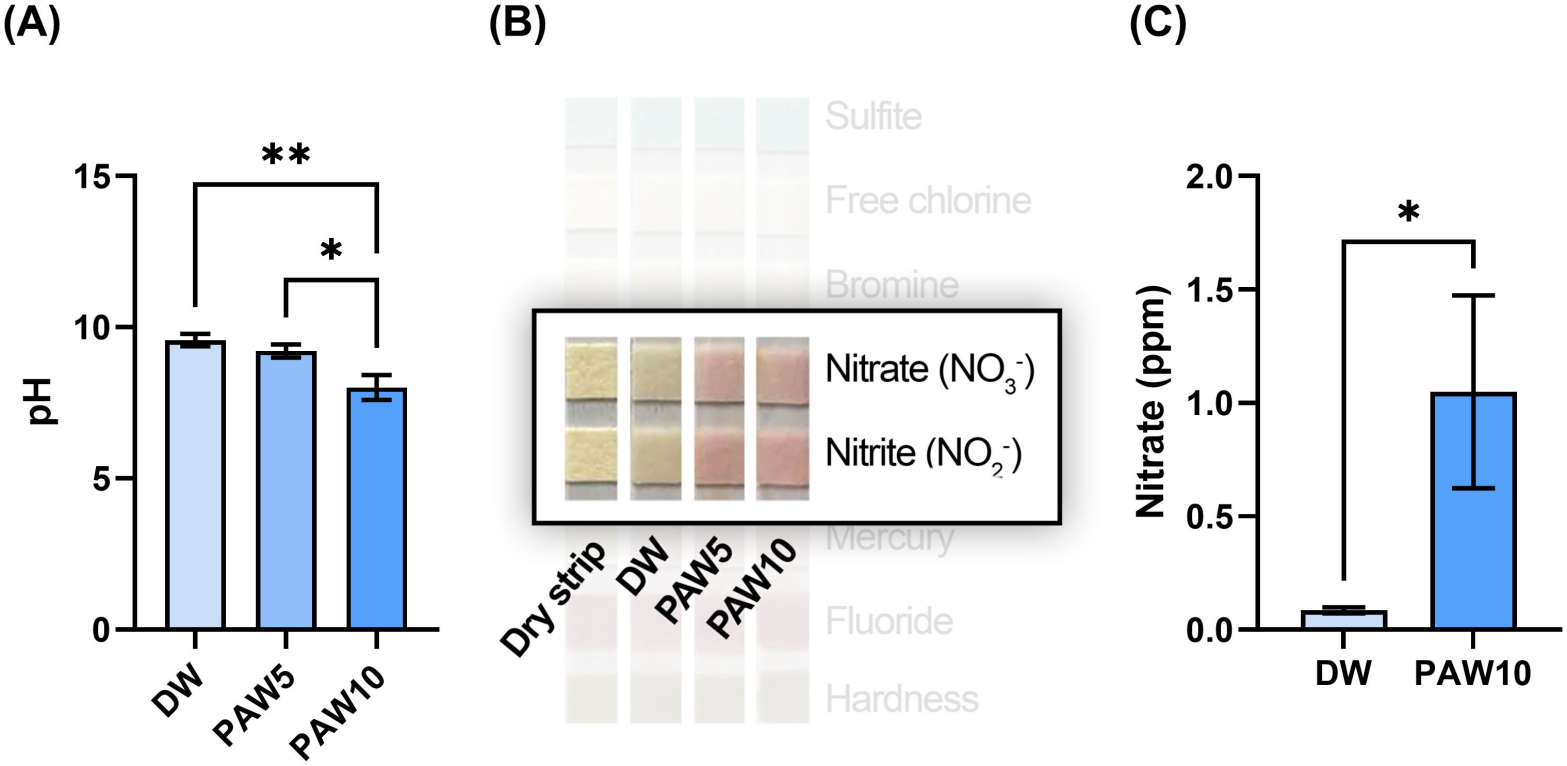
Physicochemical properties of untreated deionized water (DW) and deionized water activated with plasma for 5 min (PAW5) and 10 min (PAW10): **(A)** pH, **(B)** presence of nitrogen species as determined through colorimetric test strips, and **(C)** nitrate content. All experiments were performed in five replicates (n=10) and error bars represent the standard error. Means with * (p<0.05) or ** (p<0.01) are significantly different from each other.

Untreated deionized water was shown to have a basic pH of 9.57 ± 0.47 (**Figure 4A**). The alkalinity of this starting material, which also served as the control, may be due to the leaching of alkali metal oxides and alkali earth metal oxides from the borosilicate glass container into the water. These components are intentionally added to glass products to impart chemical and heat resistance (Hasanuzzaman et al., 2016). However, under high temperatures beyond 100 °C, such as during autoclaving, glass can be corroded via an ion-exchange reaction, where alkali ions in the glass diffuse into the water. During this process, H^+^ ions in the water fill the vacant sites in the glass; consequently, the concentration of H^+^ in the water decreases, and the pH of the solution increases (Lee et al., 2023).

After 10-min exposure of deionized water to the Ar plasma, a significant decrease in pH was observed, from 9.57 ± 0.47 in the untreated control to 8.01 ± 0.83 (p = 0.007). The decrease in pH values can be associated with increased H^+^ concentrations from additional reactions of plasma species in the water (Equations 12–19).

(12) $$O^{*}+N_{2}\to NO+N^{*}$$

(13) $$N^{*}+O_{2}\to NO+O^{*}$$

(14) $$NO+O^{*}\to NO_{2}$$

(15) $$2NO_{2}\to N_{2}O_{4}$$

(16) $$N_{2}O_{4}+H_{2}O\to HNO_{3}+HNO_{2}$$

(17) $$3NO_{2}+H_{2}O\to 2H^{+}+2NO_{3}^{-}+NO$$

(18) $$HNO_{3}\to H^{+}+NO_{3}^{-}$$

(19) $$HNO_{2}\to H^{+}+NO_{2}^{-}$$

Simply put, plasma treatment of water for 10 min at a gap distance of 1.5 cm enables the formation of RNS such as nitric oxide (NO), nitric dioxide (NO_2_), and dinitrogen tetroxide (N_2_O_4_) (Equations 12–15). These species can hydrolyze to ultimately release H^+^ ions that cause the observed pH reduction (Equations 16–19). This process occurs as species in the plasma, such as O_2_, N_2_, and excited atoms of oxygen (O*) and nitrogen (N*), react further in water to form NO (Equations 12, 13) (Marcinauskas et al., 2024; Klimek and Piercey, 2024). NO may then undergo more reactions to produce NO_2_, N_2_O_4_, nitrous acid (HNO_2_) and nitric acid (HNO_3_) (Equations 14–16) (Taaca et al., 2023). Alongside the hydrolytic products of NO_2_ (Equation 17), the latter two acids contribute to the H^+^ ions in the water (Equations 18, 19). This is possible because plasma interactions with deionized water generate more RNS than plasma in ambient air, which is why NO was not detected in the OES results earlier (**Figure 2**).

As evident in the reactions, accumulation of H^+^ ions in PAW is accompanied by the release of nitrates (NO_3_^-^) and nitrites (NO_2_^-^) into solution (Equations 18, 19). This was confirmed through colorimetric strips that indicate the presence of NO_3_^-^ and NO_2_^-^ (**Figure 4B**), as well as through quantitation using a NO_3_^--^selective electrode (**Figure 4C**). PAW10 was shown to contain 1.05 ± 0.74 ppm of NO_3_^-^, which is significantly higher than the 0.09 ± 0.02 ppm found in the control DW (p = 0.043).

### 3.4 Germination and growth characteristics

**Figure 5:**
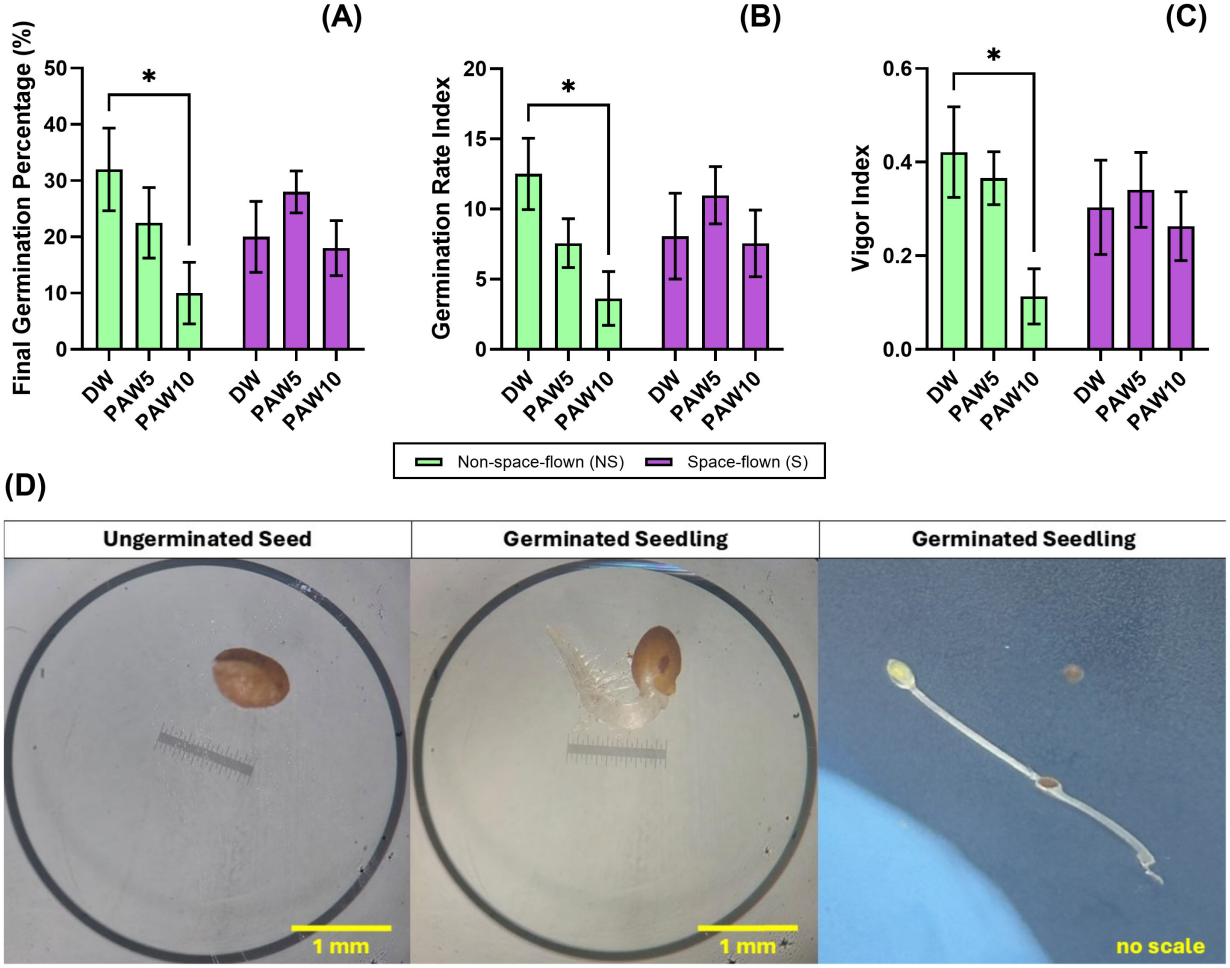
Characteristics of PAW-treated spearmint seeds after 14-day germination: **(A)** final germination percentage, **(B)** germination rate index, **(C)** and vigor index. All experiments were performed in five replicates (n=10) and error bars represent the standard error. Means with * (p<0.05) are significantly different from each other based on pairwise comparisons. **(D)** Criteria for germination in calculating final germination percentage, where a seed is considered to have germinated when the emerging radicle is visually longer than the longest axis of the seed.

Without PAW treatment (DW), NS and S seeds had no significant differential response in any of the three germination and growth characteristics (FGP, GRI, and VI) (**Figures 5A–C**). This indicates that prior space exposure does not have a measurable impact on the overall seed performance under unstressed basal conditions. TZ staining reinforces this, revealing consistent mean percent viabilities between S seeds (78.33 ± 6.66%) and the NS cohort (76.00 ± 7.94%; p = 0.7169). Hence, spaceflight history does not affect initial seed quality — a finding that aligns with previous reports on the viability of space-flown rice seeds (Sugimoto et al., 2016; Zeng et al., 2021). This uniformity explains the identical baseline performance of both types of seeds under DW, and at the same time, ensures that any changes upon PAW treatment are unobscured by underlying viability differences.

Two-way ANOVA revealed that the main effect of space exposure on all three characteristics of germination and growth (FGP, GRI, and VI) was not significant (p = 0.917, p = 0.623, p = 0.973, respectively). Thus, direct statistical comparisons based on the main effects of space-exposure history are unlikely to be meaningful. In contrast, the application of PAW treatment appeared to show modest significance in the outcomes of the three characteristics (p = 0.085, p = 0.130, p = 0.067, respectively). Interestingly, employing pairwise comparisons between treatments within the same cohort (within either S only or NS only) showed that the spearmint seed’s response to PAW depends on whether it had been previously flown in space (**Figure 5**). Specifically, the application of PAW10 resulted in a significant decrease in the NS cohort’s potential to break dormancy (**Figure 5A**), the rate at which seeds germinate into a seedling (**Figure 5B**), and the amount of biomass produced after 14-day incubation (**Figure 5C**). This is in direct contrast to the S cohort, whose germination and growth did not seem to suffer from the apparently inhibitory effects of PAW10. Overall, the data suggest that while spaceflight alone does not alter dormancy release, germination rate, and seed vigor, it confers a change in how the seed responds to PAW. This differential response to PAW10 treatment led us to investigate the underlying changes that occur in spearmint seeds following space exposure.

### 3.5 Gas chromatography-electron ionization mass spectrometry (GC-EI/MS) analysis

To provide molecular insights on the observations from the germination assay, GC-EI/MS was performed on ethanolic extracts of both ungerminated seeds and germinated seedlings after PAW10 treatment. The two major volatile components found to be present in all samples are 2,4-di-tert-butylphenol (2,4-DTBP) and methyl palmitate (MP) (**Supplementary Table 1**). Other detected compounds were not present in all samples.

2,4-DTBP is a lipophilic phenol that commonly occurs in the volatile profile of many seed plants. It has been previously reported in other members of the Lamiaceae family (Zhao et al., 2020). While the compound is known to exhibit a variety of bioactivities — such as anti-inflammatory, insecticidal, nematocidal, antibacterial, antifungal, and allelopathic — it is largely appreciated for its antioxidant properties. This has enabled its wide use in various consumer products such as fuels, pharmaceuticals, and stabilizers (Hoang and Park, 2024). In plants, its biological role is scarcely described, but one study by Romero-Correa et al. (2014) has reported its function in avocado. They demonstrated that 2,4-DTBP acts as a defense compound against pathogens by inhibiting their ROS production. This compound’s antioxidant activity is supported *in vitro* through thiobarbituric acid reactive substances (TBARS) and 2,2′-diphenyl-1-picrylhydrazyl (DPPH) assays, which estimate lipid peroxidation and free radical scavenging, respectively (Yoon et al., 2006; Ayswarya et al., 2022; Aravinth et al., 2023). On the other hand, MP is a fatty acid methyl ester (FAME), which is mainly known for its insecticidal and acaricidal activities (Liu et al., 2009; Wang et al., 2009). To the best of our knowledge, there are no reports of MP as an antioxidant defense molecule in plant stress response.

**Figure 6:**
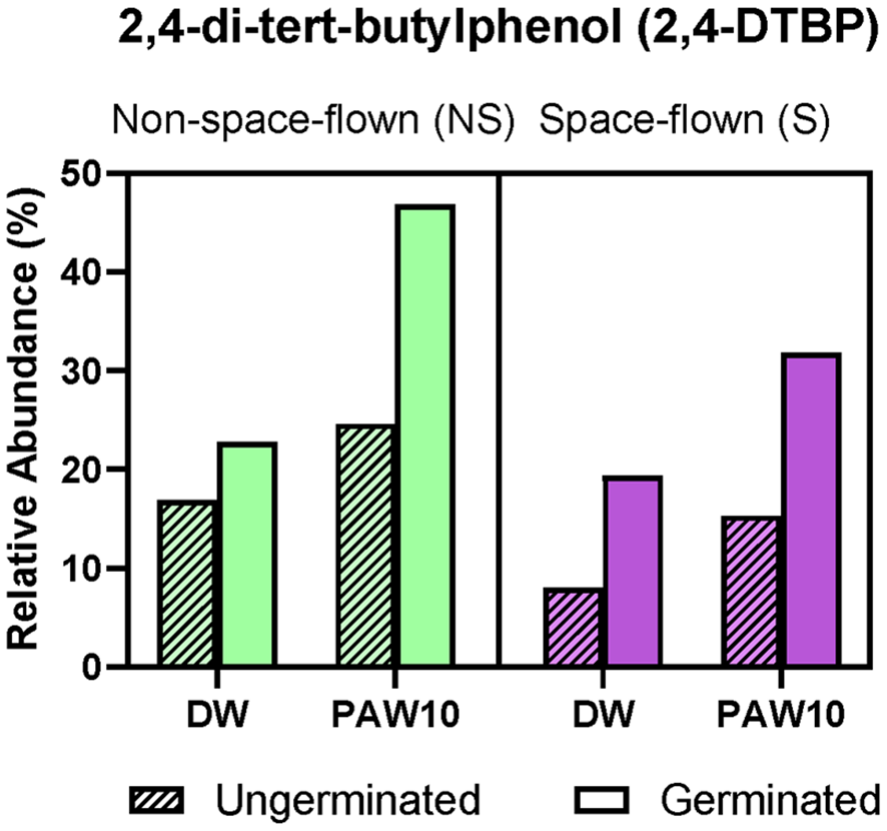
Relative abundance of 2,4-di-tert-butylphenol (2,4-DTBP) in PAW10-treated germinated seedlings and ungerminated seeds with (S) and without (NS) space-exposure history.

Given that phenolics are typically utilized in plants as a non-enzymatic antioxidant — as opposed to FAMEs — the antioxidant response of spearmint upon PAW10 treatment is better assessed through its 2,4-DTBP content (**Figure 6**). It can be observed that 2,4-DTBP is present at lower levels in S seeds and seedlings relative to their NS counterparts. Moreover, both NS and S seedlings generally contain a higher content of 2,4-DTBP compared to the ungerminated seed state. Then, upon application of PAW10, 2,4-DTBP content increases in both NS and S cohorts compared to when only DW is used. This is especially pronounced in NS samples, where a greater increase can be observed from DW to PAW10 in the germinated seedlings. These results point to a potential role of space exposure in modulating the defense response of spearmint against stress, which, in this case, appears to have been caused by the application of PAW10.

## 4 Discussion

The seed is the simple reproductive unit of higher plants; it consists of an embryonic axis and one or two cotyledons. In mature seeds, the embryo is encased by storage tissue, which presents a barrier to the radicle’s protrusion during germination (Steinbrecher and Leubner-Metzger, 2018). As such, the embryo is kept in a dormant, desiccated state, where low water content minimizes molecular diffusion and mobility, and results in the loss of the integrity of membranes and their protein components (El-Maarouf-Bouteau, 2022). Consequently, water uptake begins the process of breaking seed dormancy by restoring cellular machinery involved in respiration and hormone synthesis, activating signaling pathways that lead to the elongation of the embryonic axis, loosening of the cell wall, and programmed cell death of layers above the embryo. This facilitates the rupture of the seed coat and underlying tissues, which favors root protrusion. At this point, the seed has germinated, and the seedling is established (**Figure 5D**).

Different RONS from plasma treatment can help facilitate the germination process and subsequent growth through mechanisms that can be ascribed to four main manifestations: (a) seed coat modification, (b) increase in growth parameters, (c) metabolism, and (d) stress resistance (Waskow et al., 2021). Plasma components can erode the lipid layer of the seed surface to enhance permeability and consequently improve water uptake (Billah et al., 2020). This effect has been shown to increase characteristics like germination rate, root length, shoot length, and seed vigor. At the molecular level, reactive species can increase the content of antioxidant enzymes and other metabolites, which equip plants with the capacity to grow under stressful conditions. In particular, RONS impose oxidative stress on the seed, which, at optimal levels, can favor the oxidative pentose phosphate pathway (PPP). This in turn, provides reducing power and ribose-5-phosphate, a key precursor for nucleotide synthesis and thereby, cell division and growth (Mujahid et al., 2020). Furthermore, RONS can trigger plant defense by upregulating expression of genes involved in the phenylpropanoid pathway, which is linked to the increase of phenolic compounds in the plant. These phytochemicals have a natural role in radiation shielding to protect against membrane lipid peroxidation and cellular DNA damage (Musgrave, 2002; Tepfer et al., 2012). RONS also regulate the expression of histone modification genes, thereby indirectly regulating various antioxidant, phytohormone, and stress resistance-related genes (Shelar et al., 2022). These biological activities are typically ascribed to NOx and H_2_O_2_, as these are long-lived species that can be transported through the plant cell membranes easily (Kučerová et al., 2021). While NOx production has been confirmed (**Figures 4B, C**), H_2_O_2_ is likely to form in solution since highly reactive OH radicals from Equations 3, 4, and 6 can (a) recombine in the gas phase prior to transfer to the bulk liquid (Equation 20), or (b) dissolve into the liquid then recombine (Equation 21) (Taaca et al., 2023).

(20) $$OH{•}_{\left(g\right)}+OH{•}_{\left(g\right)}\to H_{2}O_{2\;\left(g\right)}\to H_{2}O_{2\;\left(aq\right)}$$

(21) $$OH{•}_{\left(aq\right)}+OH{•}_{\left(aq\right)}\to H_{2}O_{2\;\left(aq\right)}$$

While indirect plasma treatment can simulate the aforementioned effects, the present study showed that the application of PAW10 hindered germination and growth of NS seeds, but not S seeds (**Figure 5**). This inhibitory action of PAW treatments has been previously reported in literature (Fan et al., 2020; Lee et al., 2020; Ka et al., 2021). Given that PAW10 and DW differed by their NO_3_^-^ content (**Figure 4C**), it is possible that this species may have been present in concentrations that exceeded the tolerance limit of spearmint. We suggest that at a discharge time of 10 min, NO_3_^-^ (and possibly other RONS) may have been introduced in excess. With growing evidence that PAW regulates gene expression and phytohormonal synthesis via redox signaling (Zambon et al., 2020; Gul et al., 2026), an uncontrolled surplus of RONS — as suggested in the present study — disrupts homeostasis such that nitro-oxidative damage subverts critical physiological processes and results in poor seed performance.

The reduction in FGP and GRI in the NS group, both of which relate to different facets of germination, could be attributed to the generation of plasma species and consequent decrease in pH in water. Among the different RONS in PAW, of importance is NO_3_^-^. This molecule is reduced to NO· when taken up by plants, which in turn modulates the hormones involved in seed dormancy such as abscisic acid, gibberellins, and indoleacetic acid (Tuan et al., 2018; Fan et al., 2020; Chuesaard et al., 2023; Wang et al., 2023b). Furthermore, NO· can interact with other ROS such as superoxide (O_2_·^-^) in plant cells, producing a powerful oxidant, peroxynitrite (ONOO^-^). This short-lived species induces nitration of tyrosine residues in proteins involved in regulating phytohormones, as well as causes oxidative and nitrosative changes in lipids and DNA (Arasimowicz-Jelonek and Floryszak-Wieczorek, 2011). Altogether, the actions of ONOO^-^ are reported to be associated with abiotic stress (Corpas et al., 2006). Also, NO· has been reported to inhibit the activity of antioxidants, as well as regulate genes involved in plant defense, oxidative stress responses, and hormone interplay to metabolism and development (Molassiotis and Fotopoulos, 2011; Timilsina et al., 2022).

Excess NO_3_^-^ may also be an important contributor to reduced germination vigor, as observed in the decrease in VI in the NS group. In the study performed by Ka et al. (2021), root lengths of thale cress (*Arabidopsis thaliana*) were reduced after 15 days of planting when treated with PAW19 and PAW40 (water treated for a discharge time of 19 and 40 min, respectively). They attributed this to the NO_3_^--^mediated regulation of genes involved in root development. Specifically, at excessive concentrations of NO_3_^-^ due to prolonged activation of water, COBL9, XTH9, and XTH17 genes were downregulated, resulting in decreased root cell expansion and hair initiation. Hence, further investigation on genes related to root and shoot development can help elucidate which pathways are involved in the toxic mechanism of excess NO_3_^-^ against plant growth.

While the discussion so far is limited to NO_3_^-^, the complex chemistry of PAW is also characterized by other RONS that were not quantified (such as NO_2_^-^ and H_2_O_2_), which can be detrimental when present in excess amounts (Oke, 1966; Cleemput and Samater, 1995; Dat et al., 2000; Gadjev et al., 2008). Just like NO_3_^-^, NO_2_^-^ can also be reduced to NO· via the NO_3_-NO_2_-NO pathway and inflict the same effects described earlier (Timilsina et al., 2022). It has also been previously described that the exogenous application of high amounts of NO_2_^-^ can reduce shoot and root growths in *Zea mays*. This was attributed to the degradation of lignin components in plant cell walls by the conjugate acid, HNO_2_ (Bolker, 1965; Lee, 1979). Meanwhile, high concentrations of H_2_O_2_ can inhibit root elongation and act as a source of O_2_·^-^ for the production of toxic ONOO^-^ (Mukherjee and Corpas, 2023).

In any case, plants are capacitated to mitigate the oxidative and nitrosative stress from RONS through the coordination of antioxidant defenses (Kapoor et al., 2019). This involves the dynamic regulation of enzymatic and non-enzymatic components (Cannea and Padiglia, 2025). Enzymatic antioxidants, which represent the first line of defense, include superoxide dismutase (SOD), catalase (CAT), ascorbate peroxidase (APX), etc. SODs, as the primary antioxidant defense enzyme, catalyze the conversion of O_2_·^-^ into H_2_O_2_, which is then decomposed by CAT or APX into water and oxygen. This enzymatic network maintains high levels of ascorbate and active glutathione (GSH), which are both non-enzymatic antioxidants. Other plant non-enzymatic antioxidants include secondary metabolites like phenolics. These complement the enzymatic system by terminating oxidation chain reactions by scavenging a broader range of reactive species (Rao et al., 2025).

In the present study, PAW10 treatment increased the 2,4-DTBP content of seedlings compared to the seed state (**Figure 6**). This was expected since PAW10 introduces the plant to RONS, and in response, 2,4-DTBP may be synthesized to scavenge these species (Yoon et al., 2006; Ayswarya et al., 2022; Aravinth et al., 2023). Notably, PAW10 treatment resulted in a comparatively larger fold-increase in 2,4-DTBP content in germinated seedlings than in the ungerminated seeds. This alludes to the normal response of the metabolically active plant to the nitro-oxidative stress induced by PAW10. While this is the case for both NS and S samples, it appears that the antioxidant defense response in the Earth-based NS controls was insufficient to wholly protect the plant, resulting in the decrease in germination and growth characteristics (**Figure 5**). These observations are in direct contrast to the S samples, where PAW10 had no effect on their germination and growth. This alone may imply a higher tolerance of S samples to nitro-oxidative stress, as conferred by prior exposure to space. However, this protective mechanism might not be due to the presence of 2,4-DTBP, but rather something else. While the assumption is that PAW-induced stress is accompanied by the greater production of antioxidant phenolics, PAW10 did not increase 2,4-DTBP content from seed to seedling to the same extent as in NS controls (**Figure 6**).

**Figure 7:**
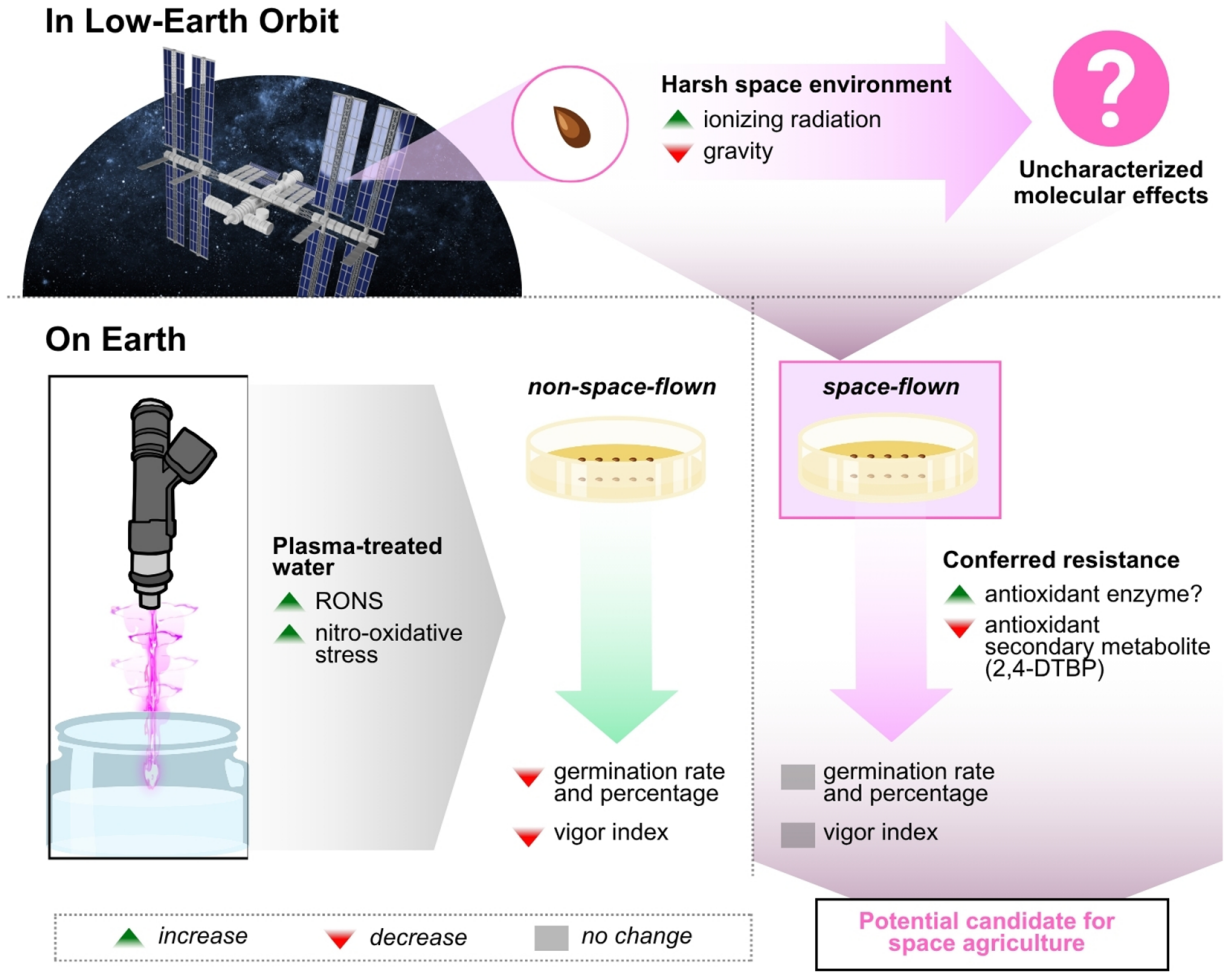
Summary diagram of the effect of spaceflight on the germination and growth of *Mentha spicata* L. seeds: The microgravity and high levels of ionizing radiation in the space environment result in molecular changes in a seed, which thereby influence their growth when cultivated. In the case of spearmint, non-space-flown seeds respond to PAW-induced nitro-oxidative stress poorly, as evidenced in reduced germination and growth characteristics. On the other hand, space-flown seeds exhibit stress tolerance as PAW treatment did not negatively impact seed performance. Simultaneously, space-flown spearmint seeds have a lower amount of 2,4-DTBP, an antioxidant secondary metabolite. The conferred resistance might be due to an enzymatic stress response that remains unknown in the current study.

We speculate that prior space exposure altered the stress response of spearmint (**Figure 7**). This has been demonstrated before in similar reports on the tomato plant. In Dzhos et al.’s study, space-flown tomato seeds resulted in higher-yielding plants with lower content of phenolics and increased antioxidant activity compared to Earth-based seeds (Dzhos et al., 2021). This was described to have potentially arisen from increased antioxidant enzyme activity, as supported by another study on the same plant (Lu et al., 2009). Preference for antioxidant defense is further exemplified by how tomato plants have been shown to favor non-enzymatic mechanisms over enzymatic counterparts upon the application of a different stimulus such as UV-A/B irradiation (Mariz-Ponte et al., 2018).

The observed enhancement of stress resistance in seeds following spaceflight has been demonstrated to be a long-term effect. In the same work by Dzhos et al., space-flown tomato seeds grew into mature plants that exhibited better tolerance to diseases in field conditions (Dzhos et al., 2021). Taking this further, these space-induced effects can carry over to succeeding generations in the tomato plant, as reported by another study (Lu et al., 2009). This memory retention of spaceflight stress has also been found in rice, where proteomics and metabolomics were used to elucidate that soluble sugars sense ROS accumulation and induce the reconstruction of other metabolic networks to protect the plant (Zeng et al., 2022).

Collectively, the above points reveal the possibility that a similar phenomenon occurred in spearmint seeds when they were previously flown into space for 6 months. We suspect that S samples were able to withstand the inhibitory effects of PAW10 treatment more so through an enzymatic antioxidant response rather than through non-enzymatic metabolites, although further experimentation is required to confirm whether this proposed biochemical basis indeed underlies our observations.

At the same time, it is important to note that these results do not preclude PAW as an assistive technology in space cultivation, as using other plant species and PAW generation parameters may yield different outcomes. In fact, the enhanced stress resistance of space-flown spearmint that was observed in this study possibly highlights the potential to harness the historically characterized benefits of PAW in germination and growth, without the worry of undesirable effects from the acute exposure to plasma-induced RONS.

## 5 Conclusion

Plasma-activated water that was generated at a discharge time of 10 min (PAW10) was shown to successfully introduce nitrogen species such as nitrates into water. To support this, necessary precursors were detected via OES, and the pH reduction associated with the formation of these species in water was observed. Treatment of spearmint seeds with nitrate-rich PAW10 significantly decreased germination and growth parameters in NS seeds, but did not affect S seeds. This points to the nitro-oxidative stress induced by PAW10 on the normal seed, as well as to the conferred resistance of space-flown seeds to this inhibitory effect. Nitrate toxicity was discussed as a likely factor, but the complex chemistry of PAW is also accompanied by other RONS and may also contribute to this observation. Further investigation via GC-EI/MS analysis showed a relatively greater increase in 2,4-DTBP in NS seedlings compared to S seedlings. Therefore, non-enzymatic antioxidant mechanisms via secondary metabolites may not be the underlying reason behind the observed stress resistance in space seeds. Based on similar studies on the tomato plant, we acknowledge the possibility that enzymatic components of the antioxidant defense system were favored, and we caution that additional work to test this hypothesis is still necessary. Regardless, these findings can be used to further investigate how to harness the plasticity of the plant stress response in incorporating biological components in space activities such as in BLSSs.

To build on the new perspectives raised in the present work, future studies are encouraged to determine the uptake of RONS in the developing plant at different timepoints. In addition, PAW-treated samples can be characterized further via metabolic profiling, mutational analysis, transcriptomics, and proteomics to elucidate how the enhanced stress response arises.

## Data Availability

The original contributions presented in the study are included in the article/**Supplementary Material**. Further inquiries can be directed to the corresponding author.

## References

[B1] Acharya T. R. Jang M. Lee G. J. Choi E. H. (2023). A comprehensive study on the synthesis, characteristics, and catalytic applications of submerged hydrogen-mixed argon plasma-synthesized silver nanoparticles. Curr. Appl. Phys. 56, 36–46. doi: 10.1016/j.cap.2023.09.003 38826717

[B2] Adhikari B. C. Lamichhane P. Lim J. S. Nguyen L. N. Choi E. H. (2021). Generation of reactive species by naturally sucked air in the Ar plasma jet. Results Phys. 30, 104863. doi: 10.1016/j.rinp.2021.104863 38826717

[B3] Almuslet N. A. Salih A. M. (2012). Nd Yag Laser. Ed. Dumitras D. C. (Erscheinungsort nicht ermittelbar: IntechOpen).

[B4] Arasimowicz-Jelonek M. Floryszak-Wieczorek J. (2011). Understanding the fate of peroxynitrite in plant cells – From physiology to pathophysiology. Phytochemistry 72, 681–688. doi: 10.1016/j.phytochem.2011.02.025 21429536

[B5] Aravinth A. Perumal P. Rajaram R. Dhanasundaram S. Narayanan M. Maharaja S. . (2023). Isolation and characterization of 2,4-di-tert-butyl phenol from the brown seaweed, Dictyota ciliolata and assessment of its anti-oxidant and anticancer characteristics. Biocatal. Agric. Biotechnol. 54, 102933. doi: 10.1016/j.bcab.2023.102933 38826717

[B6] Arroyo C. L. Concepcion R. Bandala (2023). Identifying the Future Trends of Bioregenerative Life Support Systems for Space Exploration (Coron, Palawan, Philippines: Institute of Electrical and Electronics Engineers), 1–6. doi: 10.1109/HNICEM60674.2023.10589031

[B7] Asghar A. H. Galaly A. R. (2021). The effect of oxygen admixture with argon discharges on the impact parameters of atmospheric pressure plasma jet characteristics. Appl. Sci. 11, 6870. doi: 10.3390/app11156870 30654563

[B8] Ayswarya S. Radhakrishnan M. Manigundan K. Gopikrishnan V. Soytong K. (2022). Antioxidant activity of 2,4-di-tert-butylphenol isolated from plant growth promoting endophytic Streptomyces KCA-1. Int. J. Agric. Technol. 18, 2343–2352. Available online at: https://li04.tci-thaijo.org/index.php/IJAT/article/view/8975

[B9] Billah M. Sajib S. A. Roy N. C. Rashid M. M. Reza M. A. Hasan M. M. . (2020). Effects of DBD air plasma treatment on the enhancement of black gram (Vigna mungo l.) seed germination and growth. Arch. Biochem. Biophys. 681, 108253. doi: 10.1016/j.abb.2020.108253 31917117

[B10] Bolker H. I. (1965). Delignification by nitrogen compounds. Action of nitrous acid on unbleached sulfate pulp. I&EC Product Res. Dev. 4, 74–79. doi: 10.1021/i360014a004

[B11] Cannea F. B. Padiglia A. (2025). Antioxidant defense systems in plants: mechanisms, regulation, and biotechnological strategies for enhanced oxidative stress tolerance. Life. 15, 1293. doi: 10.3390/life15081293 40868941 PMC12387146

[B12] Chandler J. O. Haas F. B. Khan S. Bowden L. Ignatz M. Enfissi E. M. A. . (2020). Rocket science: the effect of spaceflight on germination physiology, ageing, and transcriptome of eruca sativa seeds. Life. 10, 49. doi: 10.3390/life10040049 32344775 PMC7235897

[B1001] Chen C.-J. Li S.-Z. (2017). Investigation of a nitrogen post-discharge of an atmospheric-pressure microwave plasma torch by optical emission spectroscopy. Phys. Plasmas. 24, 033512. doi: 10.1063/1.4978948

[B13] Cheng L. Li Y. Yan J. (2025). Space biological and human survival: Investigations into plants, animals, microorganisms and their components and bioregenerative life support systems. Life Sci. Space Res. 44, 143–153. doi: 10.1016/j.lssr.2024.10.007 39864907

[B14] Chou Y.-J. Ting Y. (2023). Plasma-activated water regulated transcriptome gene expression leading to high germination and growth of mung beans. Chem. Biol. Technol. Agric. 10, 146. doi: 10.1186/s40538-023-00497-2 38164791

[B15] Chuesaard T. Peankid P. Thaworn S. Jaradrattanapaiboon A. Veerana M. Panngom K. (2023). Different effects of reactive species generated from chemical donors on seed germination, growth, and chemical contents of oryza sativa L. Plants 12, 765. doi: 10.3390/plants12040765 36840122 PMC9966467

[B16] Cleemput O. Samater A. H. (1995). Nitrite in soils: accumulation and role in the formation of gaseous N compounds. Fertilizer Res. 45, 81–89. doi: 10.1007/BF00749884 30311153

[B17] Corpas F. J. Barroso J. B. Carreras A. Valderrama R. Palma J. M. Del Río L. A. (2006). “ Nitrosative stress in plants: A new approach to understand the role of NO in abiotic stress,” in Nitric Oxide in Plant Growth, Development and Stress Physiology. Eds. Lamattina L. Polacco J. C. ( Springer Berlin Heidelberg, Berlin, Heidelberg), 187–205. doi: 10.1007/7089_2006_091

[B18] Dat J. Vandenabeele S. Vranová E. Van Montagu M. Inzé D. Van Breusegem F. (2000). Dual action of the active oxygen species during plant stress responses. Cell. Mol. Life. Sci. (CMLS) 57, 779–795. doi: 10.1007/s000180050041 10892343 PMC11147059

[B19] De Micco V. Amitrano C. Mastroleo F. Aronne G. Battistelli A. Carnero-Diaz E. . (2023a). Plant and microbial science and technology as cornerstones to Bioregenerative Life Support Systems in space. NPJ Microgravity 9, 69. doi: 10.1038/s41526-023-00317-9 37620398 PMC10449850

[B20] De Micco V. Aronne G. Caplin N. Carnero-Diaz E. Herranz R. Horemans N. . (2023b). Perspectives for plant biology in space and analogue environments. NPJ Microgravity 9, 67. doi: 10.1038/s41526-023-00315-x 37604914 PMC10442387

[B21] Dutuit O. Carrasco N. Thissen R. Vuitton V. Alcaraz C. Pernot P. . (2013). Critical review of N, N^+^, N^+^_2_, N^++^, and N^++^_2_ main production processes and reaction sof relevance to titan’s atmosphere. ApJS 204, 20. doi: 10.1088/0067-0049/204/2/20

[B22] Dzhos E. Golubkina N. Antoshkina M. Kondratyeva I. Koshevarov A. Shkaplerov A. . (2021). Effect of spaceflight on tomato seed quality and biochemical characteristics of mature plants. Horticulturae 7, 89. doi: 10.3390/horticulturae7050089 30654563

[B23] Ehrlich J. W. Massa G. Wheeler R. Gill T. R. Quincy C. Roberson L. . (2017). “ Plant growth optimization by vegetable production system in HI-SEAS analog habitat”, in: AIAA SPACE and Astronautics Forum and Exposition (Orlando, FL: American Institute of Aeronautics and Astronautics). doi: 10.2514/6.2017-5143

[B24] El Boukhari R. Fatimi A. (2025). “ The innovative potential of key mentha species: an assessment based on patent analysis”, in: The 3rd International Electronic Conference on Diversity: Biodiversity of Animals, Plants and Microorganisms ( MDPI). 5. doi: 10.3390/blsf2024039005

[B25] Elhindi K. M. Dewir Y. H. Asrar A.-W. Abdel-Salam E. El-Din A. S. Ali M. (2016). Improvement of seed germination in three medicinal plant species by plant growth regulators. horts 51, 887–891. doi: 10.21273/HORTSCI.51.7.887

[B26] El-Maarouf-Bouteau H. (2022). The seed and the metabolism regulation. Biology 11, 168. doi: 10.3390/biology11020168 35205035 PMC8869448

[B27] Fan L. Liu X. Ma Y. Xiang Q. (2020). Effects of plasma-activated water treatment on seed germination and growth of mung bean sprouts. J. Taibah Univ. Sci. 14, 823–830. doi: 10.1080/16583655.2020.1778326 37339054

[B28] Gadjev I. Stone J. Gechev T. (2008). “ Chapter 3: programmed cell death in plants,” in International Review of Cell and Molecular Biology. Eds. Bourne G. Jeon K. Jarvik J. Danielli J. Friedlander M. ( Elsevier), 87–144. doi: 10.1016/S1937-6448(08)01403-2 19081535

[B29] Gaur N. Patenall B. L. Ghimire B. Thet N. T. Gardiner J. E. Le Doare K. E. . (2023). Cold atmospheric plasma-activated composite hydrogel for an enhanced and on-demand delivery of antimicrobials. ACS Appl. Mater. Interfaces 15, 19989–19996. doi: 10.1021/acsami.3c01208 37040527 PMC10141252

[B30] Graves D. B. Bakken L. B. Jensen M. B. Ingels R. (2019). Plasma activated organic fertilizer. Plasma Chem. Plasma Process. 39, 1–19. doi: 10.1007/s11090-018-9944-9 30311153

[B31] Gul K. Mumtaz S. Al-Saif A. M. (2026). Harnessing plasma-activated water and salicylic acid synergy to enhance germination and seedling growth of tomato via redox and hormonal modulation. Plasma Chem. Plasma Process. 46, 16. doi: 10.1007/s11090-025-10637-1 30311153

[B32] Hasanuzzaman M. Rafferty A. Sajjia M. Olabi A.-G. (2016). “ Properties of glass materials,” in Reference Module in Materials Science and Materials Engineering ( Elsevier). doi: 10.1016/B978-0-12-803581-8.03998-9

[B33] Hoang N. M. H. Park K. (2024). Applications of tert-butyl-phenolic antioxidants in consumer products and their potential toxicities in humans. Toxics 12, 869. doi: 10.3390/toxics12120869 39771084 PMC11679822

[B34] Jha N. K. Sharma P. Gupta S. (2023). Sustainable life support systems for human space exploration. Int. J. Enhanced Res. Science Technology Eng. 12, 114–125.

[B35] Jia H. Fujiwara H. Kondo M. Kuraseko H. (2008). Optical emission spectroscopy of atmospheric pressure microwave plasmas. J. Appl. Phys. 104, 054908. doi: 10.1063/1.2975345

[B36] Ka D. H. Priatama R. A. Park J. Y. Park S. J. Kim S. B. Lee I. A. . (2021). Plasma-Activated Water Modulates Root Hair Cell Density via Root Developmental Genes in Arabidopsis thaliana L. Appl. Sci. 11, 2240. doi: 10.3390/app11052240 30654563

[B37] Kabashkin I. Glukhikh S. (2023). Life cycle cost model for life support systems of crewed autonomous transport for deep space habitation. Appl. Sci. 13, 8213. doi: 10.3390/app13148213 30654563

[B38] Kapoor D. Singh S. Kumar V. Romero R. Prasad R. Singh J. (2019). Antioxidant enzymes regulation in plants in reference to reactive oxygen species (ROS) and reactive nitrogen species (RNS). Plant Gene 19, 100182. doi: 10.1016/j.plgene.2019.100182 38826717

[B39] Klimek A. Piercey D. G. (2024). Nitrogen fixation via plasma-assisted processes: mechanisms, applications, and comparative analysis—A comprehensive review. Processes 12, 786. doi: 10.3390/pr12040786 30654563

[B40] Kramida A. Ralchenko Y. Reader J. NIST ASD Team (2024). NIST Atomic Spectra Database (Ver. 5.12). (Gaithersburg, MD: National Institute of Standards and Technology). doi: 10.18434/T4W30F

[B41] Kučerová K. Henselová M. Slováková Ľ. Bačovčinová M. Hensel K. (2021). Effect of plasma activated water, hydrogen peroxide, and nitrates on lettuce growth and its physiological parameters. Appl. Sci. 11, 1985. doi: 10.3390/app11051985 30654563

[B1000] Langa S. Magwaza L. S. Mditshwa A. Tesfay S. Z. (2024). Seed dormancy and germination responses of cannabis landraces to various pre-treatments. S. Afr. J. Bot. 169, 91–100. doi: 10.1016/j.sajb.2023.12.021

[B42] Lasseur C. Brunet J. de Weever H. Dixon M. Dussap G. Godia F. . (2010). MELiSSA: The European project of a closed life support system. Gravitational Space Biol. 23, 3–12. Available online at: https://www.researchgate.net/publication/253263459_Melissa_The_European_project_of_a_closed_life_support_system.

[B44] Lee R. B. (1979). The effect of nitrite on root growth of barley and maize. New Phytol. 83, 615–622. doi: 10.1111/j.1469-8137.1979.tb02293.x 40046247

[B43] Lee J. E. Kim E. Hwang J. B. Choi J. C. Lee J. K. (2023). Flake formation and composition in soda-lime-silica and borosilicate glasses. Heliyon 9, e16333. doi: 10.1016/j.heliyon.2023.e16333 37292333 PMC10245155

[B45] Lee Y. K. Lim J. Hong E. J. Kim S. B. (2020). Plasma-activated water regulates root hairs and cotyledon size dependent on cell elongation in Nicotiana tabacum L. Plant Biotechnol. Rep. 14, 663–672. doi: 10.1007/s11816-020-00641-6 30311153

[B46] Li L. Nikiforov A. Xiong Q. Britun N. Snyders R. Lu X. . (2013). OH radicals distribution in an Ar-H2O atmospheric plasma jet. Phys. Plasma 20, 093502. doi: 10.1063/1.4820945

[B47] Liu Y.-B. Wang Y.-N. Shi G.-L. Ren J.-J. Zhao L. Du J. . (2009). “ Acaricidal activity of methyl palmitate to T. cinnabarinus”, in: 2009 3rd International Conference on Bioinformatics and Biomedical Engineering (Beijing: IEEE), 1–4. doi: 10.1109/ICBBE.2009.5162263

[B48] Lu J. Xue H. Pan Y. Kan S. Liu M. Nechitailo G. S. (2009). Effect of spaceflight duration of subcellular morphologies and defense enzyme activities in earth-grown tomato seedlings propagated from space-flown seeds. Russ. J. Phys. Chem. B 3, 981–986. doi: 10.1134/S1990793109060190

[B49] Mahdavian K. (2025). Integrating biotechnology and microbial synergies in Mentha-based phytoremediation for sustainable pollution mitigation. Ecol. Front. 46, 450–458. doi: 10.1016/j.ecofro.2025.10.016 38826717

[B50] Marcinauskas L. Kavaliauskas Ž. Jonynaitė K. Uscila R. Aikas M. Keršulis S. . (2024). The influence of voltage on gliding arc discharge characteristics, the composition of air plasma, and the properties of BG-11 medium. Appl. Sci. 14, 2135. doi: 10.3390/app14052135 30654563

[B51] Mariz-Ponte N. Mendes R. J. Sario S. Ferreira De Oliveira J. M. P. Melo P. Santos C. (2018). Tomato plants use non-enzymatic antioxidant pathways to cope with moderate UV-A/B irradiation: A contribution to the use of UV-A/B in horticulture. J. Plant Physiol. 221, 32–42. doi: 10.1016/j.jplph.2017.11.013 29223880

[B52] Marshall Porterfield D. Tulodziecki D. Wheeler R. Davis Cross M. K. Monje O. Rothschild L. J. . (2025). Critical investments in bioregenerative life support systems for bioastronautics and sustainable lunar exploration. NPJ Microgravity 11, 57. doi: 10.1038/s41526-025-00518-4 40818983 PMC12357894

[B53] Mei M. Qiu Y. He Y. Bucker H. Yang C. H. (1994). Mutational effects of space flight on Zea mays seeds. Adv. Space Res. 14, 33–39. doi: 10.1016/0273-1177(94)90447-2 11539968

[B54] Mieno T. Kamo T. Hayashi D. Shoji T. Kadota K. (1996). Efficient production of O+ and O- ions in a helicon wave oxygen discharge. Appl. Phys. Lett. 69, 617–619. doi: 10.1063/1.117925

[B55] Molassiotis A. Fotopoulos V. (2011). Oxidative and nitrosative signaling in plants: two branches in the same tree? Plant Signal. Behav. 6, 210–214. doi: 10.4161/psb.6.2.14878 21325889 PMC3121980

[B56] Mujahid Z. Tounekti T. Khemira H. (2020). Cold plasma treatment to release dormancy and improve growth in grape buds: a promising alternative to natural chilling and rest breaking chemicals. Sci. Rep. 10, 2667. doi: 10.1038/s41598-020-59097-x 32060299 PMC7021807

[B57] Mukherjee S. Corpas F. J. (2023). H_2_O_2_, NO, and H_2_S networks during root development and signalling under physiological and challenging environments: Beneficial or toxic? Plant Cell Environ. 46, 688–717. doi: 10.1111/pce.14531 36583401 PMC10108057

[B58] Musgrave M. E. (2002). Seeds in space. Seed Sci. Res. 12, 1–17. doi: 10.1079/SSR200193 36007395

[B59] Musielok J. Wiese W. L. Veres G. (1995). Atomic transition probabilities and tests of the spectroscopic coupling scheme for N i. Phys. Rev. A. 51, 3588–3597. doi: 10.1103/PhysRevA.51.3588 9912025

[B60] Naseem I. Khan M. A. Habib U. Rana R. M. Qasim M. Alwahibi M. S. . (2025). Morphological profiling and DNA barcoding revealed genetic diversity and phylogeny of Mentha species cultivated in Pakistan. Genet. Resour. Crop Evol. 72, 2977–2995. doi: 10.1007/s10722-024-02140-x 30311153

[B61] Nelson M. Pechurkin N. S. Allen J. P. Somova L. A. Gitelson J. I. (2010). “ Closed ecological systems, space life support and biospherics,” in Environmental Biotechnology. Eds. Wang L. K. Ivanov V. Tay J.-H. ( Humana Press, Totowa, NJ), 517–565. doi: 10.1007/978-1-60327-140-0_11

[B62] Neuhäuser M. Bärwulf S. Hilgers H. Lugscheider E. Riester M. (1999). Optical emission spectroscopy studies of titanium nitride sputtering on thermoplastic polymers. Surf. Coat. Technol. 116–119, 981–985. doi: 10.1016/S0257-8972(99)00214-5

[B63] Northway S. Brown R. Fry R. (1980). Atomic nitrogen spectra in the argon inductively coupled plasma (ICP). Appl. Spectrosc. - Appl. Spectrosc. 34, 338–348. doi: 10.1366/0003702804730484

[B64] Northway S. J. Fry R. C. (1980). Atomic oxygen spectra in the argon inductively coupled plasma (ICP). Appl. Spectrosc. 34, 332–338. doi: 10.1366/0003702804730501

[B65] Oke O. L. (1966). Nitrite toxicity to plants. Nature 212, 528–528. doi: 10.1038/212528a0 37880705

[B66] Pearse R. Gaydon A. (1941). The Identification of Molecular Spectra. 1st Edn (London: Chapman & Hall Ltd).

[B67] Priatama R. A. Pervitasari A. N. Park S. Park S. J. Lee Y. K. (2022). Current advancements in the molecular mechanism of plasma treatment for seed germination and plant growth. IJMS 23, 4609. doi: 10.3390/ijms23094609 35562997 PMC9105374

[B68] Qu H. Zhao G. Wang Y. Liang L. Zhang L. Liu W. . (2019). Plasma-exposure-induced mobility enhancement of liTFSI-doped spiro-OMeTAD hole transport layer in perovskite solar cells and its impact on device performance. Materials 12, 3142. doi: 10.3390/ma12193142 31561493 PMC6803871

[B69] Rao M. J. Duan M. Zhou C. Jiao J. Cheng P. Yang L. . (2025). Antioxidant defense system in plants: reactive oxygen species production, signaling, and scavenging during abiotic stress-induced oxidative damage. Horticulturae 11, 477. doi: 10.3390/horticulturae11050477 30654563

[B70] Ren W. Zhang Y. Deng B. Guo H. Cheng L. Liu Y. (2010). Effect of space flight factors on alfalfa seeds. Afr. J. Biotechnol. 9, 7273–7279. doi: 10.5897/AJB10.532

[B71] Robinson R. Johnston S. Feldman E. Houde M. Kurkowski S. Mast A. . (2024). Seed viability testing guide for common wetland plant species (Logan, UT: Utah State University’s Wetland Ecology and Restoration Laboratory and Utah State University Extension). Available online at: https://digitalcommons.usu.edu/extension_curall/2389/.

[B72] Romero-Correa M. T. Villa-Gómez R. Castro-Mercado E. García-Pineda E. (2014). The avocado defense compound phenol-2,4-bis (1,1-dimethylethyl) is induced by arachidonic acid and acts via the inhibition of hydrogen peroxide production by pathogens. Physiol. Mol. Plant Pathol. 87, 32–41. doi: 10.1016/j.pmpp.2014.05.003 38826717

[B73] Saktioto S. Zakky F. Fardinata R. Abdullah H. Y. Fadhali M. M. (2023). Equilibrium of argon plasma particles at high pressure. Sintechcom 4, 21–30. doi: 10.59190/stc.v4i1.254

[B74] Saqib S. Ullah F. Naeem M. Younas M. Ayaz A. Ali S. . (2022). Mentha: nutritional and health attributes to treat various ailments including cardiovascular diseases. Molecules 27, 6728. doi: 10.3390/molecules27196728 36235263 PMC9572119

[B75] Schneider C. A. Rasband W. S. Eliceiri K. W. (2012). NIH Image to ImageJ: 25 years of image analysis. Nat. Methods 9, 671–675. doi: 10.1038/nmeth.2089 22930834 PMC5554542

[B76] Shelar A. Singh A. V. Dietrich P. Maharjan R. S. Thissen A. Didwal P. N. . (2022). Emerging cold plasma treatment and machine learning prospects for seed priming: a step towards sustainable food production. RSC Adv. 12, 10467–10488. doi: 10.1039/D2RA00809B 35425017 PMC8982346

[B77] Shelepova O. V. Yuorieva N. O. Olecknovich L. S. Konovalova L. N. Gulevich A. A. Baranova E. N. (2025). The impact of clonal micropropagation and acclimatization methods on the productivity and essential oil quality of three mentha cultivars. Agronomy 15, 2870. doi: 10.3390/agronomy15122870 30654563

[B78] Song M.-Y. Cho H. Karwasz G. P. Kokoouline V. Nakamura Y. Tennyson J. . (2021). Cross sections for electron collisions with H2O. J. Phys. Chem. Ref. Data 50, 023103. doi: 10.1063/5.0035315 42028584

[B79] Steinbrecher T. Leubner-Metzger G. (2018). Tissue and cellular mechanics of seeds. Curr. Opin. Genet. Dev. 51, 1–10. doi: 10.1016/j.gde.2018.03.001 29571069

[B80] Sugimoto M. Oono Y. Kawahara Y. Gusev O. Maekawa M. Matsumoto T. . (2016). Gene expression of rice seeds surviving 13- and 20-month exposure to space environment. Life Sci. Space Res. 11, 10–17. doi: 10.1016/j.lssr.2016.10.001 27993188

[B81] Taaca K. L. M. Prieto E. I. Vasquez M. R. (2023). Atmospheric pressure plasma treatment of chitosan-acrylic acid blends. J. Vac. Sci. Technol. B 41, 034001. doi: 10.1116/6.0002335 42438036

[B82] Tafrihi M. Imran M. Tufail T. Gondal T. A. Caruso G. Sharma S. . (2021). The wonderful activities of the genus mentha: not only antioxidant properties. Molecules 26, 1118. doi: 10.3390/molecules26041118 33672486 PMC7923432

[B83] Tepfer D. Zalar A. Leach S. (2012). Survival of plant seeds, their UV screens, and nptII DNA for 18 months outside the international space station. Astrobiology 12, 517–528. doi: 10.1089/ast.2011.0744 22680697

[B84] Timilsina A. Dong W. Hasanuzzaman M. Liu B. Hu C. (2022). Nitrate–nitrite–nitric oxide pathway: A mechanism of hypoxia and anoxia tolerance in plants. IJMS 23, 11522. doi: 10.3390/ijms231911522 36232819 PMC9569746

[B85] Tuan P. A. Kumar R. Rehal P. K. Toora P. K. Ayele B. T. (2018). Molecular mechanisms underlying abscisic acid/gibberellin balance in the control of seed dormancy and germination in cereals. Front. Plant Sci. 9. doi: 10.3389/fpls.2018.00668 29875780 PMC5974119

[B87] Wang J. Cheng J.-H. Sun D.-W. (2023b). Enhancement of wheat seed germination, seedling growth and nutritional properties of wheat plantlet juice by plasma activated water. J. Plant Growth Regul. 42, 2006–2022. doi: 10.1007/s00344-022-10677-3 35668726 PMC9152647

[B88] Wang Y. N. Wang H. X. Shen Z. J. Zhao L. L. Clarke S. R. Sun J. H. . (2009). Methyl palmitate, an acaricidal compound occurring in green walnut husks. ec 102, 196–202. doi: 10.1603/029.102.0128 19253637

[B86] Wang B. Zhu D. Ding R. Gao B. Yan R. Li C. . (2023a). Observations on arcing on the metal plasma-facing components in EAST. Nucl. Mater. Energy 34, 101318. doi: 10.1016/j.nme.2022.101318 38826717

[B89] Waskow A. Howling A. Furno I. (2021). Mechanisms of plasma-seed treatments as a potential seed processing technology. Front. Phys. 9, 617345. doi: 10.3389/fphy.2021.617345

[B90] Wenåker I. (1990). The spectrum of singly ionized oxygen, O II. Phys. Scr. 42, 667–684. doi: 10.1088/0031-8949/42/6/008

[B91] Yoon M.-A. Jeong T.-S. Park D.-S. Xu M.-Z. Oh H.-W. Song K.-B. . (2006). Antioxidant effects of quinoline alkaloids and 2,4-di-tert-butylphenol isolated from scolopendra subspinipes. Biol. Pharm. Bull. 29, 735–739. doi: 10.1248/bpb.29.735 16595909

[B92] Yousefian S. Esmaeili F. Lohrasebi T. (2023). A comprehensive review of the key characteristics of the genus mentha, natural compounds and biotechnological approaches for the production of secondary metabolites. Iran. J. Biotechnol. 21, e3605. doi: 10.30498/ijb.2023.380485.3605 38269203 PMC10804064

[B93] Zaki F. S. A. Khalid K. A. Ahmed A. M. A. (2024). Mint species (Mentha longifolia and Mentha spicata L) growth, essential oil generation and chemical components are impacted by turmeric curcumin applications. Discov. Appl. Sci. 6, 160. doi: 10.1007/s42452-024-05810-8 30311153

[B94] Zambon Y. Contaldo N. Laurita R. Várallyay E. Canel A. Gherardi M. . (2020). Plasma activated water triggers plant defence responses. Sci. Rep. 10, 19211. doi: 10.1038/s41598-020-76247-3 33154510 PMC7644721

[B95] Zeng D. Cui J. Yin Y. Dai C. Zhao H. Song C. . (2022). Combining proteomics and metabolomics to analyze the effects of spaceflight on rice progeny. Front. Plant Sci. 13, 900143. doi: 10.3389/fpls.2022.900143 35800606 PMC9253829

[B96] Zeng D. Cui J. Yin Y. Xiong Y. Liu M. Guan S. . (2021). Metabolomics analysis in different development stages on SP0 generation of rice seeds after spaceflight. Front. Plant Sci. 12, 700267. doi: 10.3389/fpls.2021.700267 34276752 PMC8278407

[B97] Zhao F. Wang P. Lucardi R. D. Su Z. Li S. (2020). Natural sources and bioactivities of 2,4-di-tert-butylphenol and it’s analogs. Toxins (Basel) 12, 35. doi: 10.3390/toxins12010035 31935944 PMC7020479

[B98] Zimmermann M. W. Gartenbach K. E. Kranz A. R. (1994). First radiobiological results of LDEF-1 experiment A0015 with Arabidopsis seed embryos and Sordaria fungus spores. Adv. Space Res. 14, 47–51. doi: 10.1016/0273-1177(94)90449-9 11539984

