# Supplementary material for: Space-flown spearmint (Mentha spicata L.) seeds potentially tolerate nitro-oxidative stress from plasma-activated water
Source: Front Plant Sci. 2026 Jul 29;17:1836606. doi: 10.3389/fpls.2026.1836606 (PMC13463293; doi:10.3389/fpls.2026.1836606)
Supplement: Supplementary file 1 [file Table1.docx]

Supplementary Material

**Supplementary Table 1.** Comparison of compounds found via GC-EI/MS in the extracts of ungerminated seeds and germinated seedlings of space-flown (S) and non-space-flown (NS) *Mentha spicata* treated with PAW10. Compounds found in all samples are preceded by an asterisk (*).

| **Compound** | **RT (min)** | **Relative Abundance (%)** | | | | | | | | | |
| --- | --- | --- | --- | --- | --- | --- | --- | --- | --- | --- | --- |
|  |  | ***Ungerminated Seeds*** | | | |  | ***Germinated Seedlings*** | | | |  |
|  |  | *NS-DW* | *S-DW* | *NS-PAW10* | *S-PAW10* |  | *NS-DW* | *S-DW* | *NS-PAW10* | *S-PAW10* |  |
| Benzene, 1,3-bis(1,1-dimethylethyl)- | 15.504 |  |  |  |  |  |  | 2.418% |  | 1.593% |  |
| *2,4-di-tert-butylphenol | 30.915 | 16.901% | 8.000% | 24.593% | 15.317% |  | 22.764% | 19.365% | 46.846% | 31.824% |  |
| Nonadecane | 44.044 |  |  |  | 1.715% |  |  |  | 0.326% |  |  |
| 2-Bromotetradecane | 44.311 | 2.356% |  |  |  |  |  |  |  |  |  |
| Decane, 2,3,5,8-tetramethyl- | 44.317 |  | 0.763% | 2.753% |  |  | 4.171% |  |  | 2.765% |  |
| Retinal | 53.595 |  | 0.790% |  |  |  |  |  |  |  |  |
| 2-Piperidinone, N-[4-bromo-n-butyl]- | 54.870 |  |  |  |  |  |  |  |  | 1.044% |  |
| 1,2-15,16-diepoxyhexadecane | 58.472 |  |  | 0.435% |  |  |  |  |  |  |  |
| Benzene, (1-methyldodecyl)- | 58.649 |  | 9.935% |  |  |  |  |  |  |  |  |
| Tetradecane, 2,6,10-trimethyl- | 59.478 | 9.963% |  | 4.193% | 7.226% |  | 11.065% |  |  |  |  |
| Octadecane, 1-chloro- | 59.492 |  | 3.191% |  |  |  |  |  | 11.610% | 9.167% |  |
| *Methyl palmitate | 60.241 | 19.797% | 7.563% | 16.781% | 10.849% |  | 16.671% | 4.372% | 8.747% | 11.847% |  |
| 7-Hexadecenal, (Z)- | 60.728 |  |  |  |  |  |  |  |  | 0.054% |  |
| tert-Hexadecanethiol | 61.910 |  |  |  | 0.986% |  |  |  |  |  |  |
| n-Hexadecanoic acid | 62.084 |  |  |  |  |  |  | 2.797% |  |  |  |
| 7-Methyl-Z-tetradecen-1-ol acetate | 64.033 | 0.258% |  |  |  |  | 2.127% |  |  |  |  |
| Cholestan-3-ol, 2-methylene-, (3β,5α)- | 67.102 |  | 0.668% |  | 0.132% |  |  |  |  |  |  |
| 9,12-Octadecadienoyl chloride, (Z,Z)- | 68.945 | 3.104% |  |  |  |  |  |  |  |  |  |
| 12-Methyl-E,E-2,13-octadecadien-1-ol | 69.084 |  |  | 2.938% |  |  |  |  |  |  |  |
| 11-Octadecenoic acid, methyl ester | 69.130 |  |  |  |  |  |  | 1.210% |  |  |  |
| cis-Vaccenic acid | 69.208 |  |  |  | 1.434% |  |  |  |  |  |  |
| 10-Octadecenoic acid, methyl ester | 69.224 |  |  |  |  |  | 5.771% |  |  |  |  |
| 1-Dodecanol, 3,7,11-trimethyl- | 69.238 |  |  | 1.750% |  |  |  |  |  |  |  |
| 9-Octadecenoic acid (Z)-, methyl ester | 69.282 | 6.756% |  | 2.088% |  |  |  |  |  |  |  |
| Heptadecanoic acid, 16-methyl-, methyl ester | 70.594 | 18.928% | 6.548% | 14.970% |  |  | 16.846% | 4.403% |  | 16.388% |  |
| Methyl stearate | 70.654 |  |  |  | 14.156% |  |  |  | 5.526% |  |  |
| Heptacosanoic acid, methyl ester | 70.838 |  |  | 7.685% |  |  |  |  |  |  |  |
| Dodecanoic acid, 2,3-bis(acetyloxy)propyl ester | 70.854 | 3.133% |  |  |  |  |  |  |  |  |  |
| Oleic Acid | 71.576 |  |  |  |  |  |  | 1.351% |  |  |  |
| 2-methylhexacosane | 72.464 |  |  | 1.299% |  |  |  |  |  |  |  |
| Octadecanoic acid | 72.729 |  |  |  |  |  |  | 13.641% |  |  |  |
| Ethanol, 2-(octadecyloxy)- | 76.799 |  |  |  | 1.224% |  | 3.384% |  |  |  |  |
| Methoxyacetic acid, 2-tetradecyl ester | 79.857 | 3.561% |  | 1.082% |  |  |  |  |  |  |  |
| Eicosane, 2-methyl- | 79.857 |  |  |  |  |  |  |  | 4.034% |  |  |
| Octadecanoic acid, 2-hydroxy-1,3-propanediyl ester | 84.476 |  |  |  |  |  |  | 0.897% |  |  |  |
| 1,3-Benzenedicarboxylic acid, bis(2-ethylhexyl) ester | 99.465 |  | 0.098% | -0.011% |  |  |  |  |  |  |  |
| Ethyl iso-allocholate | 106.689 |  |  |  |  |  |  | 0.535% |  |  |  |
| Lup-20(29)-en-3-ol, acetate, (3β)- | 108.070 |  |  |  |  |  |  | 1.154% |  |  |  |
| Stigmastan-3,5-diene | 109.073 |  |  |  |  |  |  | 7.918% |  |  |  |
| β-Sitosterol | 115.736 |  |  |  |  |  |  | 5.567% |  |  |  |
